# Single-support serial isomorphous replacement phasing

**DOI:** 10.1107/S2059798322003977

**Published:** 2022-05-09

**Authors:** Nicolas Foos, Mahmoud Rizk, Max H. Nanao

**Affiliations:** a Structural Biology, European Synchrotron Radiation Facility, 71 Avenue des Martyrs, 38000 Grenoble, France

**Keywords:** single isomorphous replacement, serial crystallography, genetic algorithms, microcrystallography, machine learning

## Abstract

The use of single isomorphous replacement (SIR) has become less widespread due to difficulties in sample preparation and the identification of isomorphous native and derivative data sets. Non-isomorphism becomes even more problematic in serial experiments, because it adds natural inter-crystal non-isomorphism to heavy-atom-soaking-induced non-isomorphism. Here, a method that can successfully address these issues (and indeed can benefit from differences in heavy-atom occupancy) and additionally significantly simplifies the SIR experiment is presented. A single heavy-atom soak into a microcrystalline slurry is performed, followed by automated serial data collection of partial data sets. This produces a set of data collections with a gradient of heavy-atom occupancies, which are reflected in differential merging statistics. These differences can be exploited by an optimized genetic algorithm to segregate the pool of data sets into ‘native’ and ‘derivative’ groups, which can then be used to successfully determine phases experimentally by SIR.

## Abstract 2

A simple method for serial single isomorphous replacement, which exploits natural differences in heavy-atom occupancy, is presented.

## 1. Introduction

Atomic resolution structural information is critical to our understanding of fundamental biological processes and plays an increasingly important role in the development and improvement of pharmaceuticals and chemical biology probes. Macromolecular crystallography (MX) is one of the most effective ways to obtain such information. However, MX can be limited by the phase problem (Taylor, 2003 ▸) and the necessity of growing large single crystals for data collection. Traditionally, the phasing of crystallographic data has required heavy-atom soaking or derivatization and crystal sizes of >100 µm. Working with smaller samples of 1–20 µm has many advantages, including a reduction in the time and material that are needed for crystal optimization, especially for challenging projects such as those with membrane proteins. It also offers a more uniform soaking of heavy atoms or ligands and more complete illumination in optical pump–probe experiments. The proliferation of microfocus synchrotron beamlines (Nanao *et al.*, 2022 ▸; Hasegawa *et al.*, 2013 ▸; Evans *et al.*, 2007 ▸) and advanced data-collection/analysis methods has facilitated measurements from these smaller crystals; however, radiation damage makes the collection of complete, high-quality data sets from single microcrystals extremely challenging (Holton & Frankel, 2010 ▸). The answer to this problem appears to be serial/multi-crystal approaches such as synchrotron serial crystallography (SSX), in which data from many crystals are merged to produce a single data set (Gati *et al.*, 2014 ▸; Stellato *et al.*, 2014 ▸; Botha *et al.*, 2015 ▸; Zander *et al.*, 2015 ▸; Hasegawa *et al.*, 2017 ▸). Indeed, combining serial methods with intense microbeams has allowed the boundaries of crystal size to be pushed in recent years. Multi-crystal methods do come at a significant price, however: the natural variation between crystals (‘non-isomorphism’) can degrade the quality of the final merged data sets (Giordano *et al.*, 2012 ▸), which is a particular challenge for phasing applications.

One of the earliest methods of experimental macromolecular crystallography phasing is the single isomorphous replacement (SIR) method (Crick & Magdoff, 1956 ▸; Green *et al.*, 1954 ▸), in which data are collected from both a heavy-atom-soaked crystal and an unsoaked ‘native’ crystal. Differences between the intensities are used to determine the positions of the heavy atoms, which can then be used to experimentally determine phases for the native protein data. SIR offers the advantages of potentially very large differences in intensity, which can in turn provide very large phasing powers. However, its use in multi-crystal methods is complicated by both natural and heavy-atom-induced non-isomorphism. Indeed, the differences in intensities due to non-isomorphism are often larger than the signal induced by heavy-atom binding. As a result, SIR has to date been relatively uncommon in multi-crystal experiments, and the existing work has primarily been on still image data from free-electron lasers (Botha *et al.*, 2015 ▸; Yamashita *et al.*, 2015 ▸; Nakane *et al.*, 2016 ▸; Zhang *et al.*, 2015 ▸). In addition to the problem of non-isomorphism, SIR has the practical limitation that successful SIR experiments typically require the preparation and collection of diffraction data from many samples in order to identify groups of crystals for which the heavy-atom occupancies and isomorphism are high enough while also maintaining sufficient diffraction quality. This process often consumes a significant amount of manpower and beamtime.

Spatiotemporal gradients of ligand concentrations have been simulated and shown experimentally (Cole *et al.*, 2014 ▸; Geremia *et al.*, 2006 ▸; Pandey *et al.*, 2021 ▸; Mizutani *et al.*, 2014 ▸; Schmidt, 2013 ▸). We reasoned that if a population of different heavy-atom occupancies could be established, we could use a genetic algorithm (GA)-based grouping technique (Zander *et al.*, 2016 ▸; Foos *et al.*, 2019 ▸; Cianci *et al.*, 2019 ▸) to distinguish derivative from native data sets. Indeed, here we report a method in which single heavy-atom soaks are performed followed by SSX data collection. A genetic algorithm is then used to group data sets that can be used to successfully determine phases experimentally by SIR.

## 2. Methods

### 2.1. Sample preparation

Four different kinds of protein microcrystals derivatized with different heavy atoms were analyzed. Lysozyme crystals of between 5 and 20 µm in size were grown in batch: a 40 mg ml^−1^ lysozyme solution was prepared in a solution consisting of 1.5 *M* NaCl, 0.1 *M* sodium acetate pH 4.6, 30% PEG 5000. Crystals of proteinase K, insulin and thermolysin were obtained using the hanging-drop vapor-diffusion method. Proteinase K crystals were prepared at 50 mg ml^−1^ in 50 m*M* HEPES pH 7.0 with a well solution consisting of 0.5–1.5 *M* sodium nitrate, 100 m*M* citrate pH 6.5. Insulin was dissolved to 15 mg ml^−1^ in 50 m*M* Na_2_HPO_4_ pH 10.4 with 1 m*M* EDTA pH 8.0 and crystallized in 350–450 m*M* Na_2_HPO_4_ pH 10.4, 10 m*M* EDTA. Thermolysin was prepared at 50 mg ml^−1^ in 50 m*M* MES pH 6.0 with 45% DMSO and the well solution consisted of 35%(*w*/*v*) ammonium sulfate dissolved in water; the crystallization drops were prepared by mixing the protein solution with the well solution in a 1:1 ratio (Marshall *et al.*, 2012 ▸). All crystals were obtained at 20°C. Large (100–500 Å) crystals were crushed between siliconized coverslips to obtain a range of microcrystal sizes between 5 and 20 µm. Stock solutions of Gd-HPDO3A (gadoteridol; Girard *et al.*, 2002 ▸), mercury(II) acetate, samarium(III) nitrate and sodium iodide were made in water at 25 m*M*, 20 m*M*, 5 m*M* and 1 *M*, respectively. These stocks were added to glycerol (final concentration of 25%) and well solution to obtain soaking buffers with final heavy-atom concentrations of 2 m*M*, 5 m*M*, 667 µ*M* and 400 m*M*, respectively. Microcrystalline slurries were transferred to 2 µl of these soaking solutions using 700 µm diameter micro-meshes with 10 µm openings (MiTeGen). The transfer of crystals is likely to be preferable to direct addition of heavy atoms to crystallization drops because of the competition of uncrystallized protein for heavy-atom binding. The heavy-atom soak times were 5 min, 4 min, 1 min and 30 s, respectively, based on previous experience with nonserial SIR experiments on larger crystals. Practically, soaking times can be established by setting up a sufficient quantity of slurry for multiple meshes and then removing slurry at several time points followed by harvesting on micro-meshes and flash-cooling in liquid nitrogen.

### 2.2. Data collection and merging

Data were collected on the fixed-energy ESRF beamline ID23-EH2 (Nanao *et al.*, 2022 ▸) at 14.2 keV with a PILATUS3 2M detector and MD3Up diffractometer (Maatel). Data collection was performed at 100 K in *MxCuBE* (Oscarsson *et al.*, 2019 ▸) using the *MeshAndCollect* workflow (Zander *et al.*, 2015 ▸) (Table 1 ▸). Diffraction images and metadata (*XDS* input files) have been uploaded to Zenodo under ID 5111402 (https://doi.org/10.5281/zenodo.5111402). Data were initially processed automatically using *XDS* and *Grenades* (Monaco *et al.*, 2013 ▸). The partial data set with the highest overall 〈*I*/σ(*I*)〉 was used as a reference data set for re-integration in *XDS* (Kabsch, 2010*b* ▸) in order to account for indexing ambiguity. It is interesting to note that even in well behaved test cases such as these, the range of unit-cell parameters across the entire pool of data sets is generally around 1–2%, which suggests a non-negligable amount of non-isomorphism. Indeed, in their pioneering analysis of non-isomorphism, Crick & Magdoff (1956 ▸) estimated that unit-cell changes of only 0.5% lead to 15% changes in intensities of acentric reflections at 3 Å. The merging *R* values are generally quite high when all data are merged (Table 2 ▸).

**Figure 1:**
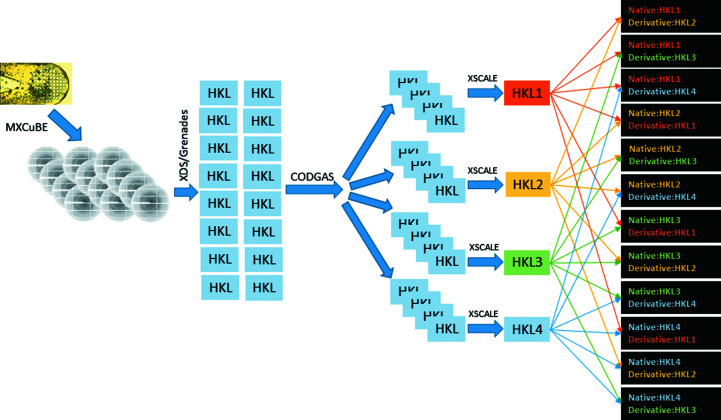
Program workflow for phasing. Data sets are collected from multiple crystals on a single support and indexed and integrated in *XDS*. These partial data sets are then submitted to *CODGAS* for grouping, and each group is submitted pairwise in both ‘directions’ to *SHELXC*/*D*/*E* for phasing.

Partial data sets were then submitted to the *CODGAS* (Zander *et al.*, 2016 ▸) genetic algorithm for separation into four groups followed by scaling and merging in *XSCALE* (Kabsch, 2010*a* ▸) (Fig. 1 ▸). The choice of the number of groups was set to a larger number than usual because of the anticipated increase in heavy-atom-induced non-isomorphism and the potential presence of both native and derivative data. The numbers of partial data sets in the native and derivative data sets are indicated in Table 2 ▸. While it would be helpful to establish a generally useful guideline for the minimum total number of partial data sets to collect in the *MeshAndCollect* workflow, this parameter is likely to vary as a function of the heavy-atom occupancy, diffraction resolution and symmetry. Indeed, Table 2 ▸ shows a dramatic range in the number of data sets comprising the final native and derivative data sets. It is likely that the total number of data sets that we collected was in great excess of what was necessary. When partial data sets are removed from the pool of lysozyme data, we found that as few as 20 partial data sets out of 67 could be used to determine the phases. Insulin and thermolysin phasing was successful with 75 out of 149 and 40 out of 53 data sets, respectively. However, the number of proteinase K data sets could only be reduced to 85 from the total of 91 collected. It should be noted, however, that the speed of the workflow makes the collection of 100 partial data sets quite rapid and there is therefore very little disadvantage in collecting a larger pool. Improvements to the GA could in principle further reduce the requirement for the total number of data sets.

Default parameters were used in the *CODGAS* target function. Execution of *CODGAS* was submitted to the ESRF SLURM cluster. Run times vary as a function of data-set parameters and cluster load and the specific machine that was allocated, but as an example execution took 133 min for the lysozyme data set with 67 total partial data sets on ten 2.4 GHz Intel Xeon E5-2680 cores. The native and derivative data sets had significantly reduced ranges of unit-cell parameters compared with the ranges of the entire pool, indicating the successful identification of isomorphic groups (Table 2 ▸).

### 2.3. Structure solution

The resultant data sets from *CODGAS* were then submitted pairwise to *SHELXC*/*D*/*E* (Sheldrick, 2010 ▸) for substructure and phase determination by SIR (without including anomalous scattering), (Fig. 1 ▸). Because only isomorphous differences were considered in this work, there is no way to determine *a priori* whether one group is native or derivative. Therefore, the SIR is performed in both ‘directions’ for each pair (Fig. 1 ▸). Phasing success was determined by visual inspection of electron-density maps in *Coot* (Emsley *et al.*, 2010 ▸) and the correlation coefficient of the automatically built partial model (‘partial CC’) in *SHELXE*. Generally, a partial CC of greater than 25% was seen as evidence of a successful structure solution, but for thermolysin some solutions with lower values (down to 18%) still yielded easily interpretable electron-density maps. Post-phasing analysis *F*_o_ − *F*_c_ difference maps were calculated for the proteinase K data set for each *CODGAS* subgroup using phases from a proteinase K model without heavy atoms. Interestingly, these maps revealed that the ‘native’ data set (group 3) was also partially derivitized (Supplementary Fig. S1), but there was apparently a large enough difference in the heavy-atom occupancies between this group and group 2 to determine the phases experimentally. The peak heights for the native and derivative were 80 and 48 standard deviations above the mean value. Analysis of *F*_o_ − *F*_c_ maps in the other systems also revealed heavy atoms in the ‘native’ data sets. Native versus derivative peak heights for thermolysin, insulin and lysozyme were 43 versus 51, 31 versus 37 and 29 versus 36 standard deviations above the mean, respectively. Merging statistics for the successful native and derivative data sets are shown in Table 2 ▸.

## 3. Results

### 3.1. *De novo* phasing

**Figure 2:**
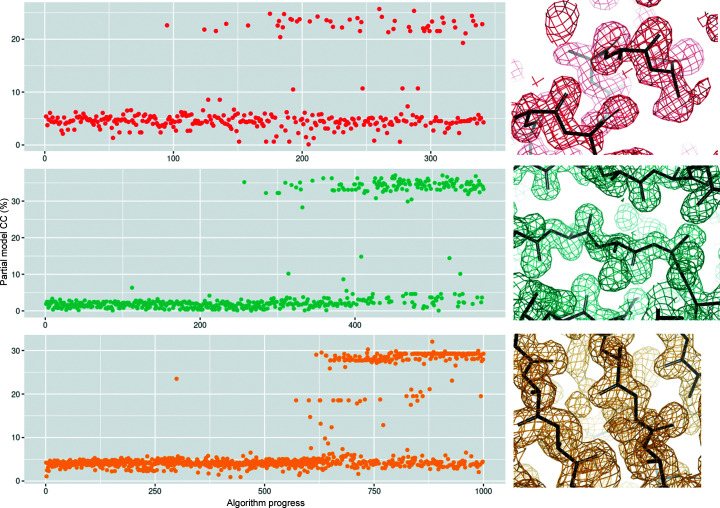
Segregation of native and isomorphous data sets can be used for SIR phasing in lysozyme Gd (upper panel), proteinase K Hg (middle panel) and thermolysin Sm (lower panel). Algorithm progress is shown on the *x* axis and the partial CC is shown on the *y* axis. Representative electron density from *SHELXE* is shown on the right at 1.5σ. The figure was produced using *ggplot*2 (https://ggplot2.tidyverse.org/), *R* (https://www.r-project.org/) and *PyMOL* (Schrödinger).

**Figure 3:**
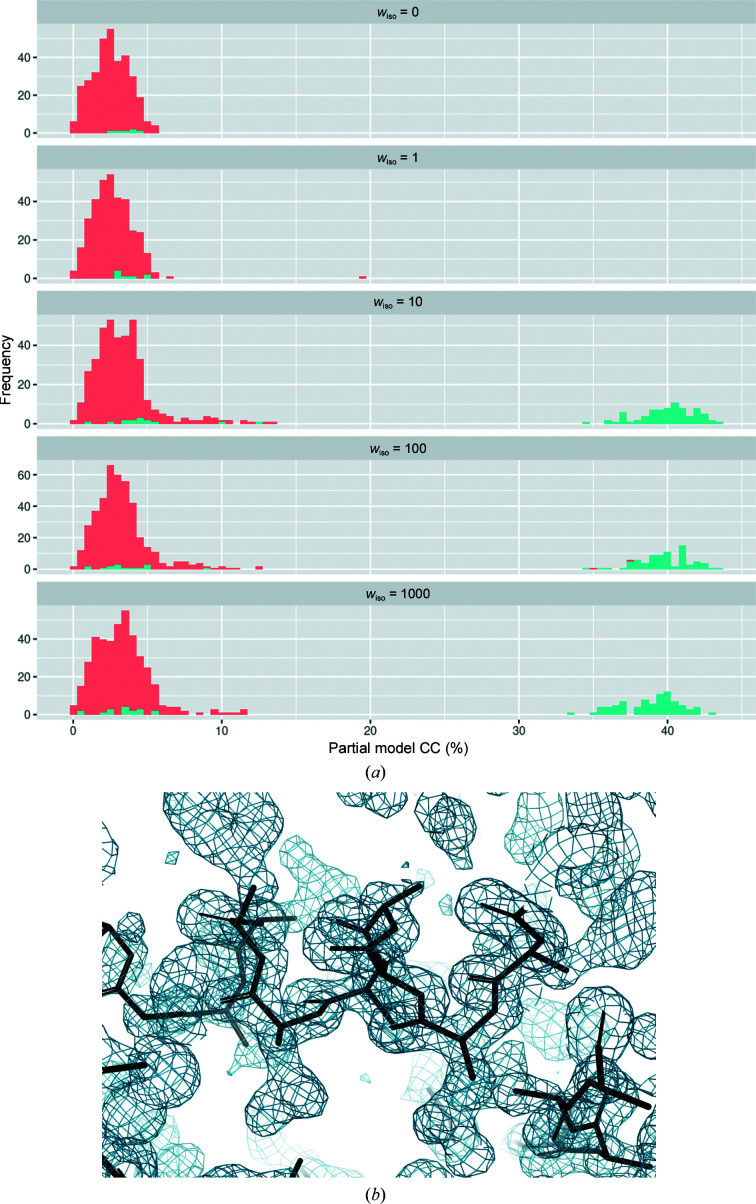
(*a*) Improvement of phasing success with the introduction of an isomorphous term in the genetic algorithm fitness function for insulin I. The frequency of the CC of the partial model is shown for *w*_iso_ of 0, 1, 10, 100, 1000 and 10 000. Average chain lengths of <11 residues per chain are shown in red and those of ≥11 are shown in cyan. (*b*) Experimental electron density from *SHELXE* contoured at 1.5σ.

Thermolysin, lysozyme and proteinase K were all solvable by this method, yielding maximum partial CCs of 32%, 25% and 37%, respectively, with easily interpretable maps (Fig. 2 ▸). Examination of intermediate generations of the GA trajectory reveals a progressive enrichment of successful phasing results as a function of algorithm progress (Fig. 2 ▸). In contrast, iodine-soaked cubic insulin was not readily solved in the same manner (Fig. 3*a* ▸, upper panel). Because the segregation of groups is dependent on both merging statistics as well as algorithmic parameters, we submitted multiple *CODGAS* runs varying both. However, changing the relative weights of the GA target function terms and the number of GA generations or the population size did not yield any improvements. While there are practical limitations to *CODGAS* parameter space, exploring it in even a fractional factorial approach can be quite time- and compute-intensive. This, coupled with the fact that not even modest improvements were observed, prompted us to adopt a different approach. We reasoned that a modification of the target function to include some metric of isomorphism might aid in group identification. We therefore introduced an additional term to the GA target function. In a classical SIR experiment, it is common to examine the merging *R* value for both the native and derivative data sets and compare it against an *R* value between the two data sets (and confirm that the absolute value of the *R* value between the data sets is not excessively high). This analysis gives the user an idea of the amount of signal and noise present in the experiment. We encoded a simple version of this heuristic analysis in a new term, based on the ratio of the intra:inter-data-set *R* values,

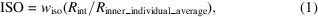

where *R*_int_ = 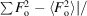 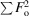 as calculated by *SHELXC*, *R*_individual_average_ is the average inner shell *R*_meas_, as calculated by *XDS*, and *w*_iso_ is the weight associated with this term. This term was added to the previously described fitness term to produce *R* + *I* + CC + *C* + *M* + ISO, where *R* = (100 − *R*_meas_ overall)*w*_R_, *I* = 〈*I*/σ(*I*)〉_overall_ *w*_*I*/σ(*I*)_, CC = 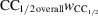 , *C* = completeness_overall_w_completeness_, *M* = multiplicity_overall_ *w*_multiplicity_ and ISO is as defined above. We then performed the GA optimization with *w*_iso_ = 1, 10, 100, 1000 and 10 000. Because GAs rely on a pseudo-random initialization of the population, in order to eliminate any effects due to different starting conditions *CODGAS* was modified in order to run with an explicitly set random-number seed. This seed is then used by the underlying GA code library (*DEAP*; https://deap.readthedocs.io/en/master/). Run in this manner, varying the *w*_iso_ term dramatically increased the number of successful structure solutions (Fig. 3 ▸). Values of *w*_iso_ of greater than 10 produced the same results, suggesting that the weighting between this term and the other GA terms is not especially critical.

## 4. Summary and outlook

Here, we apply recent analysis methods to single isomorphous replacement, resulting in a method with unique advantages. This method can be performed using data from a single heavy-atom soak and sample holder, dramatically simplifying the SIR experiment. Sample preparation is followed by data collection using existing automated workflows such as *MeshAndCollect* (Zander *et al.*, 2015 ▸). Such a data-collection strategy requires some method to separate native from derivative data sets. To this end, we have used the *CODGAS* GA, and indeed have demonstrated that such an approach can be used to identify two groups of internally isomorphous data sets and that the intensity differences between these data sets can be successfully used for *de novo* phase determination by SIR. It should be noted that we have used well behaved test systems, and it remains to be seen what the limits of this method are, particularly with respect to minimum resolution and lower symmetry.

Several improvements are already envisaged. The current target function applies only to merging statistics, but it is also possible that using metrics from downstream phasing steps could also be used. For example, an initial attempt at using *SHELXD* substructure solutions has been investigated. However, a metric of substructure correctness that is suitable for the target function has not yet been identified. The typically used CC(all) and CC(weak) metrics, for example, do not appear to offer sufficient discrimination between spurious and real solutions. Furthermore, there is a significant computational cost associated with this method. In this work, we have focused purely on isomorphous phasing, but by combining serial anomalous scattering (Melnikov *et al.*, 2017 ▸) with SIR (SIRAS) the success rate could also be improved, and this is currently being studied. The anomalous signal, where present, could also be used to establish which data set is native and which is derivative. However, strong anomalous signal is not always available, depending on the element and beamline properties.

In this work, we have largely ignored radiation-damage effects by using relatively low doses. In some cases, specific radiation damage can be used for phasing (Banumathi *et al.*, 2004 ▸; Nanao *et al.*, 2005 ▸; Schiltz *et al.*, 2004 ▸; Ravelli *et al.*, 2003 ▸; Nanao & Ravelli, 2006 ▸; de Sanctis & Nanao, 2012 ▸). This technique can be loosely viewed as an ‘inverted’ SIR experiment. We have previously shown that radiation-damage-induced phasing is possible in serial experiments (Foos *et al.*, 2018 ▸). This work employed a modified *MeshAndCollect* workflow which repeatedly collected data from the same crystals in order to obtain high- and low-dose data sets. However, it is also possible that differential radiation damage between crystals could be used in an analogous way to the gradient of heavy-atom occupancies used here. This would remove the requirement for multiple collections from the same crystals.

The suitability of cluster analysis (CA) based on correlations on intensities and or unit-cell parameters (Giordano *et al.*, 2012 ▸; Santoni *et al.*, 2017 ▸; Foadi *et al.*, 2013 ▸; Liu *et al.*, 2011 ▸) or more sophisticated approaches using *XSCALE_ISOCLUSTER* and *XDSCC*12 (Assmann *et al.*, 2020 ▸) has not yet been studied for SIR. However, it is possible that the GA and CA approaches could be complementary or indeed combined. For example, pre-grouping data with CA followed by fine-tuning in the GA could improve the separation and quality of the ‘native’ and ‘derivative’ data sets. Because the ‘native’ data sets contain some heavy atoms, there is clearly room for improvement in this regard.

Finally, while all systems were readily solved, the distribution of heavy-atom occupancies, which is related to the binding kinetics and crystal size, is likely to be a critical factor in the success of this technique. We have employed relatively gentle (short incubation time, low concentrations) heavy-atom soaking protocols in this study. However, the distribution of heavy-atom occupancies could perhaps be improved by varying the crystal sizes, beam sizes, heavy-atom concentrations and soak times. Nevertheless, we have demonstrated an extremely accessible experimental phasing protocol with associated computational analysis tools to reinvigorate the routine use of SIR in MX experiments.

## 5. Related literature

The following reference is cited in the supporting information for this article: Adams *et al.* (2010 ▸).

## Supplementary Material

Diffraction images and metadata.: https://doi.org/10.5281/zenodo.5111402

Supplementary Figure S1. DOI: 10.1107/S2059798322003977/wa5134sup1.pdf

## Figures and Tables

**Table 1:** Data-collection parameters

	Lysozyme Gd	Insulin I	Thermolysin Sm	Proteinase K Hg
Beam size (horizontal × vertical FWHM) (µm)	7 × 5	7 × 5	7 × 5	7 × 5
Photon flux (photons s^−1^)	8.3 × 10^10^	5 × 10^11^	5 × 10^11^	7 × 10^10^
Exposure per image (s)	0.1	0.03	0.01	0.03
No. of images per data set	100	100	100	100
Oscillation range per image (°)	0.1	0.1	0.1	0.1
No. of partial data sets	67	149	53	91
Ring mode, current	16 bunch, 74 mA	4 bunch, 35 mA	16 bunch, 84 mA	7/8 multibunch, 195 mA

**Table:** Values in parentheses are for the outer shell. Note that some partial data sets were not assigned to either native or derivative groups.

	Lysozyme Gd			Insulin I		
	All	Native	Derivative	All	Native	Derivative
Wavelength (A˙)	0.873	0.873	0.873	0.873	0.873	0.873
Resolution range (A˙)	39.02–1.50 (1.55–1.50)	39.02–1.50 (1.55–1.50)	39.02–1.50 (1.55–1.50)	55.47–1.60 (1.66–1.60)	55.47–1.60 (1.66–1.60)	55.47–1.60 (1.66–1.60)
Space group	*P*4_3_2_1_2	*P*4_3_2_1_2	*P*4_3_2_1_2	*I*2_1_3	*I*2_1_3	*I*2_1_3
Unit-cell average and [range]						
*a* (Å)	77.97 [76.95–78.50]	78.04 [77.53–78.23]	78.04 [77.63–78.33]	78.53 [78.06–78.74]	78.46 [78.06–78.61]	78.56 [78.36–78.74]
*b* (Å)	77.97 [76.95–78.50]	78.04 [77.53–78.23]	78.04 [77.63–78.33]	78.53 [78.06–78.74]	78.46 [78.06–78.61]	78.56 [78.36–78.74]
*c* (Å)	38.46 [37.80–38.95]	38.57 [38.02–38.95]	38.57 [37.80–38.76]	78.53 [78.06–78.74]	78.46 [78.06–78.61]	78.56 [78.36–78.74]
α (°)	90	90	90	90	90	90
β (°)	90	90	90	90	90	90
γ (°)	90	90	90	90	90	90
Total no. of reflections	912946 (86831)	328389 (31081)	467234 (44672)	1690273 (169635)	328587 (33071)	476936 (47848)
No. of unique reflections	19625 (1917)	19623 (1917)	19625 (1917)	10773 (1077)	10773 (1077)	10773 (1077)
Multiplicity	46.52 (45.30)	16.73 (16.21)	23.81 (23.30)	156.90 (157.51)	30.50 (30.71)	44.27 (44.43)
Completeness (%)	100.00 (100.00)	99.99 (100.00)	100.00 (100.00)	100.00 (100.00)	100.00 (100.00)	100.00 (100.00)
〈*I*/σ(*I*)〉	10.9 (1.3)	6.7 (0.6)	10.1 (1.1)	54.8 (12.0)	24.1 (4.5)	29.1 (6.4)
Wilson *B* factor (A˙^2^)	12.45	12.45	12.45	13.17	13.17	13.17
*R*_merge_	0.370 (7.501)	0.344 (7.020)	0.261 (4.021)	0.110 (0.956)	0.094 (0.912)	0.119 (0.989)
*R*_meas_	0.374 (7.585)	0.355 (7.248)	0.267 (4.111)	0.110 (0.959)	0.096 (0.928)	0.120 (1.000)
*R*_p.i.m._	0.054 (1.114)	0.086 (1.778)	0.054 (0.841)	0.009 (0.076)	0.018 (0.166)	0.018 (0.149)
CC_1/2_	0.998 (0.520)	0.996 (0.227)	0.999 (0.459)	1.000 (0.991)	0.995 (0.944)	0.999 (0.970)
Partial data-set statistics						
No. of partial data sets	67	24	34	149	29	42
Average completeness (%)	44.21 (42.96)	44.3 (42.53)	44.38 (43.62)	67.07 (66.93)	67.86 (67.90)	67.20 (66.27)
Average 〈*I*/σ(*I*)〉	2.72 (0.19)	2.68 (0.15)	3.19 (0.28)	7.05 (1.12)	7.19 (1.00)	6.70 (1.10)
Average *R*_meas_	0.52 (1.75)	0.33 (3.91)	0.35 (7.88)	0.09 (0.80)	0.08 (0.96)	0.11 (0.49)
Average CC_1/2_	0.92 (0.05)	0.97 (0.05)	0.95 (0.09)	0.99 (0.50)	1.00 (0.47)	0.98 (0.49)

**Table:** 

	Thermolysin Sm			Proteinase K Hg		
	All	Native	Derivative	All	Native	Derivative
Wavelength (A˙)	0.873	0.873	0.873	0.873	0.873	0.873
Resolution range (A˙)	80.74–1.60 (1.66–1.60)	80.74–1.60 (1.66–1.60)	80.74–1.60 (1.66–1.60)	57.43–1.40 (1.45–1.40)	57.43–1.40 (1.45–1.40)	57.43–1.40 (1.45–1.40)
Space group	*P*6_1_22	*P*6_1_22	*P*6_1_22	*P*4_3_2_1_2	*P*4_3_2_1_2	*P*4_3_2_1_2
Unit-cell average and [range]						
*a* (Å)	93.10 [92.24–93.48]	92.96 [92.94–93.02]	93.10 [92.93–93.20]	67.95 [67.58–68.22]	67.93 [67.76–68.03]	67.93 [67.79–68.06]
*b* (Å)	93.10 [92.24–93.48]	92.96 [92.94–93.02]	93.10 [92.93–93.20]	67.95 [67.58–68.22]	67.93 [67.76–68.03]	67.93 [67.79–68.06]
*c* (Å)	129.33 [127.60–130.88]	129.04 [129.03–129.06]	129.05 [128.84–129.29]	107.60 [106.17–108.51]	107.57 [106.89–108.12]	107.66 [107.09–107.97]
α (°)	90	90	90	90	90	90
β (°)	90	90	90	90	90	90
γ (°)	120	120	120	90	90	90
Total no. of reflections	2337994 (232519)	134562 (13566)	223412 (22425)	3190131 (301988)	772620 (72946)	561441 (53061)
No. of unique reflections	44378 (4362)	31848 (3133)	43712 (4315)	50269 (4940)	50238 (4939)	50243 (4938)
Multiplicity	52.68 (53.31)	4.23 (4.33)	5.11 (5.20)	63.46 (61.13)	15.38 (14.77)	11.17 (10.75)
Completeness (%)	100.00 (100.00)	71.76 (71.82)	98.49 (98.92)	100.00 (100.00)	99.94 (99.98)	99.95 (99.96)
〈*I*/σ(*I*)〉	10.6 (2.4)	7.2 (1.5)	6.3 (1.5)	9.9 (2.3)	6.4 (1.3)	5.0 (1.0)
Wilson *B* factor (A˙^2^)	13.87	13.87	13.87	13.20	13.20	13.20
*R*_merge_	0.436 (3.548)	0.106 (0.775)	0.141 (0.910)	0.553 (3.049)	0.362 (2.055)	0.393 (2.305)
*R*_meas_	0.441 (3.582)	0.121 (0.883)	0.157 (1.009)	0.558 (3.074)	0.375 (2.128)	0.412 (2.422)
*R*_p.i.m._	0.061 (0.486)	0.056 (0.411)	0.066 (0.423)	0.070 (0.391)	0.094 (0.544)	0.123 (0.732)
CC_1/2_	0.997 (0.790)	0.996 (0.583)	0.993 (0.618)	0.995 (0.806)	0.993 (0.570)	0.987 (0.402)
Partial data-set statistics						
No. of partial data sets	53	3	5	91	22	16
Average completeness (%)	57.82 (58.78)	68.82 (69.10)	56.52 (57.76)	48.05 (47.03)	47.49 (47.05)	50.84 (48.80)
Average 〈*I*/σ(*I*)〉	2.58 (0.38)	4.53 (0.93)	4.34 (0.96)	1.86 (0.31)	2.30 (0.40)	2.16 (0.36)
Average *R*_meas_	0.33 (4.22)	0.13 (0.84)	0.15 (1.01)	0.51 (4.68)	0.42 (1.03)	0.45 (0.33)
Average CC_1/2_	0.96 (0.16)	0.99 (0.51)	0.99 (0.46)	0.90 (0.14)	0.94 (0.18)	0.93 (0.16)
