# Supplementary material for: Single-support serial isomorphous replacement phasing
Source: Acta Crystallogr D Struct Biol. 2022 May 9;78(Pt 6):716–24. doi: 10.1107/S2059798322003977 (PMC9159287; doi:10.1107/S2059798322003977)
Supplement: Supplementary file 1 [file d-78-00716-sup1.pdf]

STRUCTURAL BIOLOGY

Volume 78 (2022)

Supporting information for article:

Single support serial isomorphous replacement phasing

Nicolas Foos, Mahmoud Rizk and Max H. Nanao

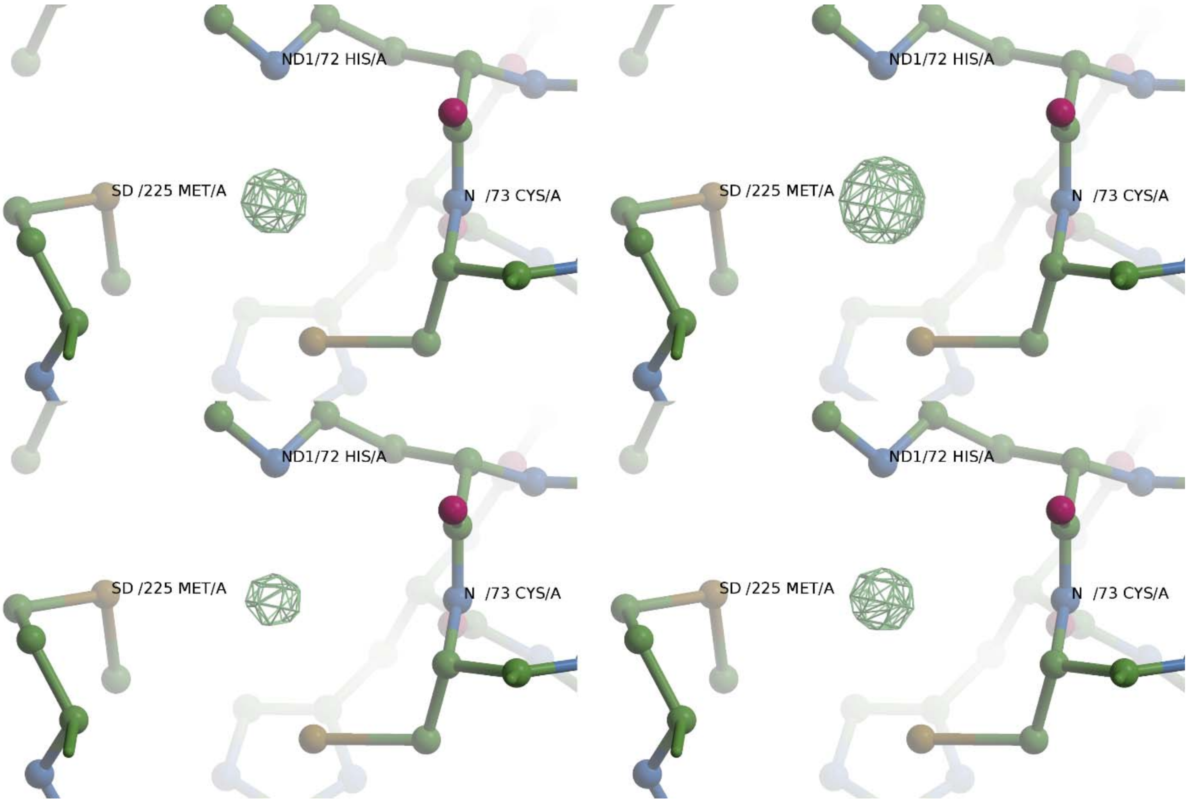

Figure S1 Fo-Fc difference maps of proteinase K for each CODGAS group (clockwise from upper left, group 1,2,3 and 4), contoured at 35 standard deviations above the mean difference density value. Maps were calculated using phenix.maps (Adams *et al.*, 2010) using default parameters. Density images were created in Coot (Emsley *et al.*, 2010).
